# The choice of endoscopic surgical approach and four steps of operation of inverted papilloma of the maxillary sinus

**DOI:** 10.1186/s12893-023-01908-9

**Published:** 2023-01-12

**Authors:** Zhengcai Lou

**Affiliations:** Department of Otorhinolaryngology, Yiwu Central Hospital, Affiliated Yiwu Hospital of Wenzhou Medical University, 699 Jiangdong Road, Yiwu, 322000 Zhejiang China

**Keywords:** Maxillary sinus, Inverted papilloma, Bone drilling, Endoscope

## Abstract

**Objective:**

The aim of this study was to determine the long-term efficacy of four steps of operation on the treatment of maxillary sinus (MS) inverted papilloma (IP).

**Methods:**

83 patients who were diagnosed with IP that originated from the MS, underwent four step procedure of attachment sites, including mucosal stripping, periosteum ablation, bone drilling and bone ablation and had postoperative follow-up of 3 years were enrolled.

**Results:**

Of the 83 patients, 59 (71.1%) patients were primary surgery and revision surgery in 24 (28.9%), single attachment was in 31(37.3%) patients and multifocal attachments in 52 (62.7%).When the numbers were not mutually exclusive, the most common origin sites of IPs were the medial wall in 54 (37.2%), lateral wall in 29 (20.0%), anterior wall in 18 (12.4%), inferior wall in 22 (15.2%), posterior in 15 (10.3%), and superior wall in 7 (4.8%). Large MMA alone was performed in 5 (6.0%), MMA combined with medial maxillectomy 76 (91.6%), and MMA combined with Caldwell-Luc approach in 2 (2.4%). No major intra- or postoperative complications were observed. The average follow-up was 41 months (range, 37–61 months). CT and endoscope showed that tumor and symptom recurrence occurred in 2 patients (2.41%). In addition, although the opening of antrostomy was closed and CT revealed the uniform soft tissue shadow and hyperostosis of MS in 11(13.3%) patients, they didn’t report any symptoms and showed well epithelization of middle meatus mucosa.

**Conclusion:**

The four steps of operations of attachment sites of MS IP, including mucosal stripping, periosteum ablation, bone drilling and bone ablation, may effectively prevent the recurrence of MS IP.

## Introduction

Sinonasal inverted papilloma (IP) is a benign tumor and constitutes about 0.5–4% of the sinonasal region tumors [1]. The distribution of attachment sites in patients with sinonasal papilloma has changed as the endoscope has enabled more detailed identification of pedicle attachment in recent years [2], the most common site of attachment was maxillary sinus (MS), followed by ethmoid sinus, frontal sinus and sphenoid sinus [1–3], the origin is 42–59% in MS [1, 2, 4]. IP can be locally aggressive; it has both the ability to recur after removal and carries a risk of converting into a malignant squamous cell carcinoma. For these reasons, the goal of surgical treatment is to completely remove the lesion by direct, visual surgery and to reduce the morbidity rate of this treatment [1, 5].The traditional external surgical approach of lateral rhinotomy with medial maxillectomy has given way to transnasal endoscopic resection. However, for anatomical reasons, the positions of the anterior and medial walls and the alveolar crypt of MS are not easily visible and manageable, therefore, it is difficult to perform complete resection using the traditional endoscopic middle meatal antrostomy (MMA) [6, 7]. In recent years, endoscopic medial maxillectomy or inferior meatal antrostomy (IMA) combined with MMA was performed to to adequately access all the wall of MS using straight instrument, thereby attempt to completely remove the tumor [1, 2, 8, 9]. In addition, endoscopic attachment-oriented surgery has became gold standard approach for the treatment of IP of MS in recent years, the approach includes the identification of the tumor attachment site, a subperiosteal dissection, and a resection or drilling of the underlying bone [1, 10, 11].

Nevertheless, some scholars reported the high recurrence rate of 3.6–10% and complications [8, 9, 12].The aim of this study was to determine the long-term efficacy of four steps of operation on the treatment of MS IPs.

## Materials and methods

### Research ethics approval

This study was reviewed and approved by the Medical Research Ethical Committee of Yiwu central hospital. Informed consent was obtained from all participants.

### Patient selection

**Fig. 1.**
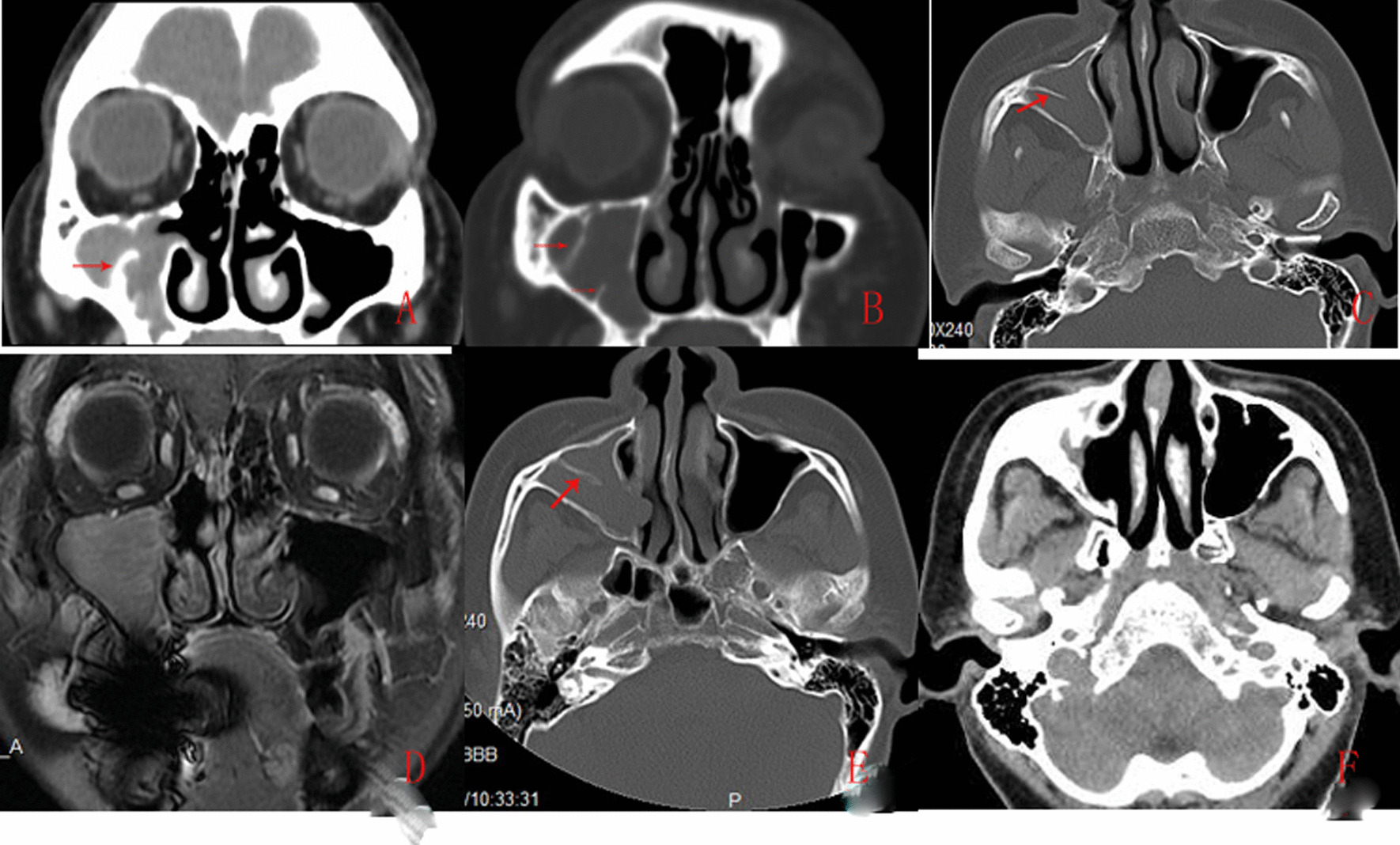
39 years-female. The first preoperative CT revealed hyperostosis and soft tissue shade of MS (**A**), the second postoperative CT revealed hyperostosis and IP recurrence at first postoperative 6 months (**B**), the second postoperative CT and MRI revealed hyperostosis and IP recurrence of MS (**C** and **D**) at second postoperative 6 months, the third preoperative CT revealed hyperostosis and IP recurrence of MS at second postoperative 10 months(**E**), the third postoperative CT revealed thickened mucosa at third postoperative 38 months (**F**). Red arrows indicated hyperostosis

We retrospectively studied the clinical data and operative records of patients with MS IP who underwent surgery from March 2015 to January 2019 in the Department of Otolaryngology. The inclusion criterias were as follows: IP was confined to MS with and without anterior ethmoid sinusitis by CT or MRI (Fig. 1) and confirmed by histologic diagnosis, and no obvious bone destruction (except the absorption of extruding bone) on CT. Demographic and clinical information were collected, including age, gender, lesion side, number of previous sinus surgeries, previous surgical procedure(s) performed, follow-up duration, and outcomes. Site of attachment was identified from operative reports.

### Surgical procedure

All patients were placed supine with the head slightly elevated; hypotensive general anesthesia was then induced. Cotton mixed with decongestants was inserted into the nose 10 min before surgery. All procedures were performed by experienced surgeons using rigid 0º, 30º, or 70º 4-mm endoscopes. A debulking of the intranasal part of the tumor was firstly performed using microdebrider. The wide MMA was performed to facilitate access to the MS and reached the satisfactory endoscopic visualization of MS in all the cases. Large tumors were debulked with a microdebrider via MMA to identify the attachment site.

The wide MMA alone was performed for the IP with the single attachment site on the superior wall, whereas combined with endoscopic medial maxillectomy (EMM) and/or Caldwell-Luc approach were respectively applied accordingly to the disease process for the IP with the attachment site on the rest of MS wall. The surgical details had been respectively described by previous authors [13–17]. Once MS was adequately exposed to view the whole tumor, which was debulked with a microdebrider to further identify the attachment site.

**Fig. 2:**
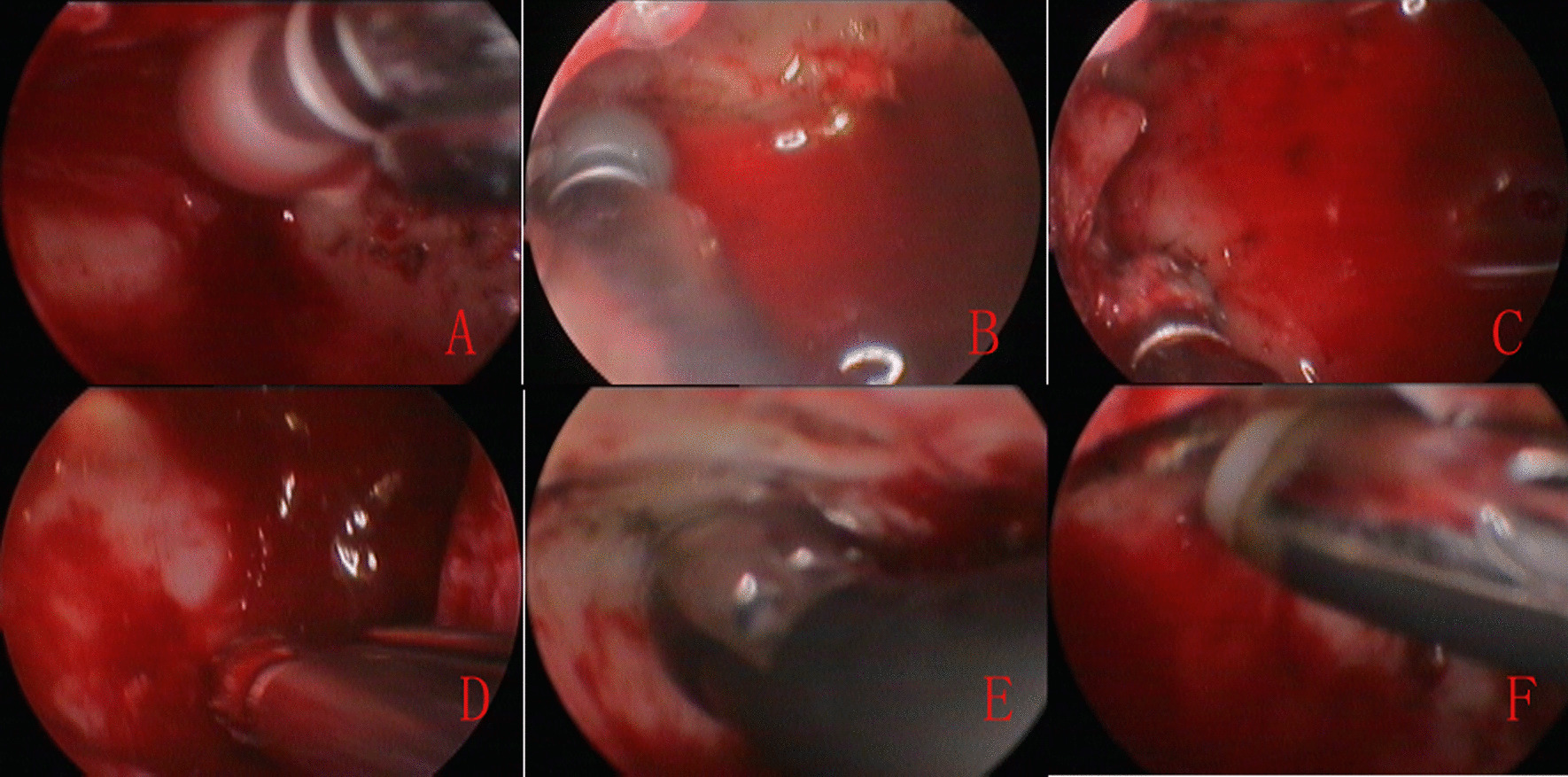
Bone drilling and ablation during the process of surgery via IMA. Anterior wall bone drilling (**A**), superior wall bone drilling (**B**), medial (**C**) and lateral wall bone drilling (**D**), bone ablation (**E** and **F**)

**Fig. 3:**
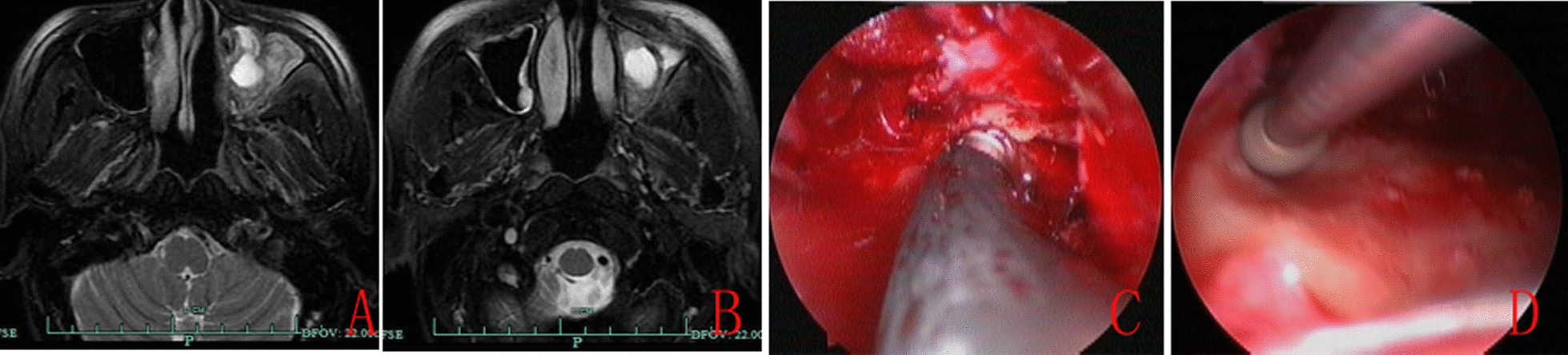
52 years-male. MRI revealed intensified soft tissue shade of left MS, the attachment sites of anterior wall (**A** and **B**), periosteum ablation (**C**), and bone drilling (**D**)

If the tumor base was identified, the mucosal stripping was performed and periosteum was ablated using radiofrequency coblation at least 2 cm around the tumor base. Otherwise, the mucosal stripping and periosteum ablation of whole MS were performed. Subsequently, drilling of the underlying bone at the site of IP attachment was applied with a diamond burr. Finally, the bone ablation was performed again to ensure that no tumor remained. Thus, the whole surgical procedure included mucosal stripping, periosteum ablation, bone drilling, and bone ablation at the site of IP attachment (Figs. 2, 3). Once the resection was completed, the mucosal flap was replaced to cover the medial maxillary wall defect and restoring the inferior meatus. All patients underwent nasal packing with Merocel, and all specimens were sent to the Department of Pathology.

### Postoperative management

Nasal packing was removed on the second postoperative day; daily nasal douching was then performed. Topical or systemic steroids were prescribed if edema of the MS mucosa was detected during the first follow-up visit. Postoperative follow-up visits were endoscopically scheduled at 1, 3, 6, 12, 24, and 36 months after surgery for all patients.

## Results

### Demographic data

In total, 83 patients with MS IPs were included in the analysis, with a mean age of 52.6 (SD: 7.9) years. Of the 83 patients, 49 patients were in male and 34 patients in female. Left nasal cavity was in 52 patients and 31 patients in right.

Of the 83 patients, 59 (71.1%) patients were primary surgery and revision surgery in 24 (28.9%). Of the 24 patients with recurrent IPs, 4 (4.8%) had previous 3 surgeries,2 surgeries in 14 (16.9%) patients, and one surgery in 6 (7.2%) patients. These previous surgery included MMA alone in 18 (75.0%) patients, Caldwell-Luc approach alone in 2 (8.3%) patients, MMA combined with IMA in 4 (16.7%) patients.

### The site of IP attachment

Among the 83 patients with IPs, CT images show hyperostosis in 21 (25.3%) patients (Fig. 1), focal osteitis in 39 (47.0%) patients, osteoneogenesis in 7(8.4%), and osteolysis in 4 (4.8%) patients (Table 1). These findings suggested that IP resulted in the pathologic changes of the underlying bone.

**Table 1:** CT showed pathologic changes of the underlying bone

Pathologic changes	N = 83
Hyperostosis	21 (25.3%)
Focal osteitis	39 (47.0%)
Osteoneogenesis	7(8.4%)
Osteolysis	4 (4.8%)

The site of attachment of IP was surgically found in all the 83 patients. Of the 83 IPs, single attachment was in 31(37.3%) patients and multifocal attachments in 52(62.7%). Comparisons of baseline characteristics between single and multiple attachment sites IPs are shown in Table 2. No statistically significant difference was noted between single and multiple attachment IPs regardless of sex, average age, and primary and revision surgery. IPs with multifocal attachments most frequently involved 2–3 walls of the sinus. IPs with single attachment predominately originated from the medial maxillary wall, while those with multiple pedicles involved primarily the medial, lateral, and anterior walls (Table 3). When the numbers were not mutually exclusive, the most common origin sites of IPs were the medial wall in 54(37.2%), lateral wall in 29 (20.0%), anterior wall in 18(12.4%), inferior wall in 22 (15.2%), posterior in 15 (10.3%), and superior wall in 7(4.8%).

**Table 2:** Comparison of baseline characteristics between single and multiple attachment sites

Variables	Single attach. (n = 31)	Multiple attach. (n = 52)	P value
Sex (male:female)	19:12	30:22	0.649^a^
Average age, years	52.8 ± 4.1	53.9 ± 2.3	0.871^b^
Primary:revision surgery	22:9	37:15	0.816^a^

^a^Chi-square test

^b^Independent Samples Test

**Table 3:** Wall of maxillary sinus involved in cases with single and multiple attachment sites

MS wall	Single attach	Multiple attach	Total
Posterior	4	11	15, 10.3%
Inferior	3	19	22, 15.2%
Lateral	5	24	29, 20.0%
Anterior	3	15	18, 12.4%
Medial	15	39	54, 37.2%
Superior	1	6	7, 4.8%
Total	31	114	145,100%

### Surgical efficacy and complications

Large MMA alone was performed in 5(6.0%), MMA combined with medial maxillectomy 76 (91.6%), and MMA combined with Caldwell-Luc approach in 2 (2.4%). No case of synchronous/metachronous squamocellular carcinoma was noted at the histological examination of the specimens. No major intra- or postoperative complications were observed in this series.

**Fig. 4:**
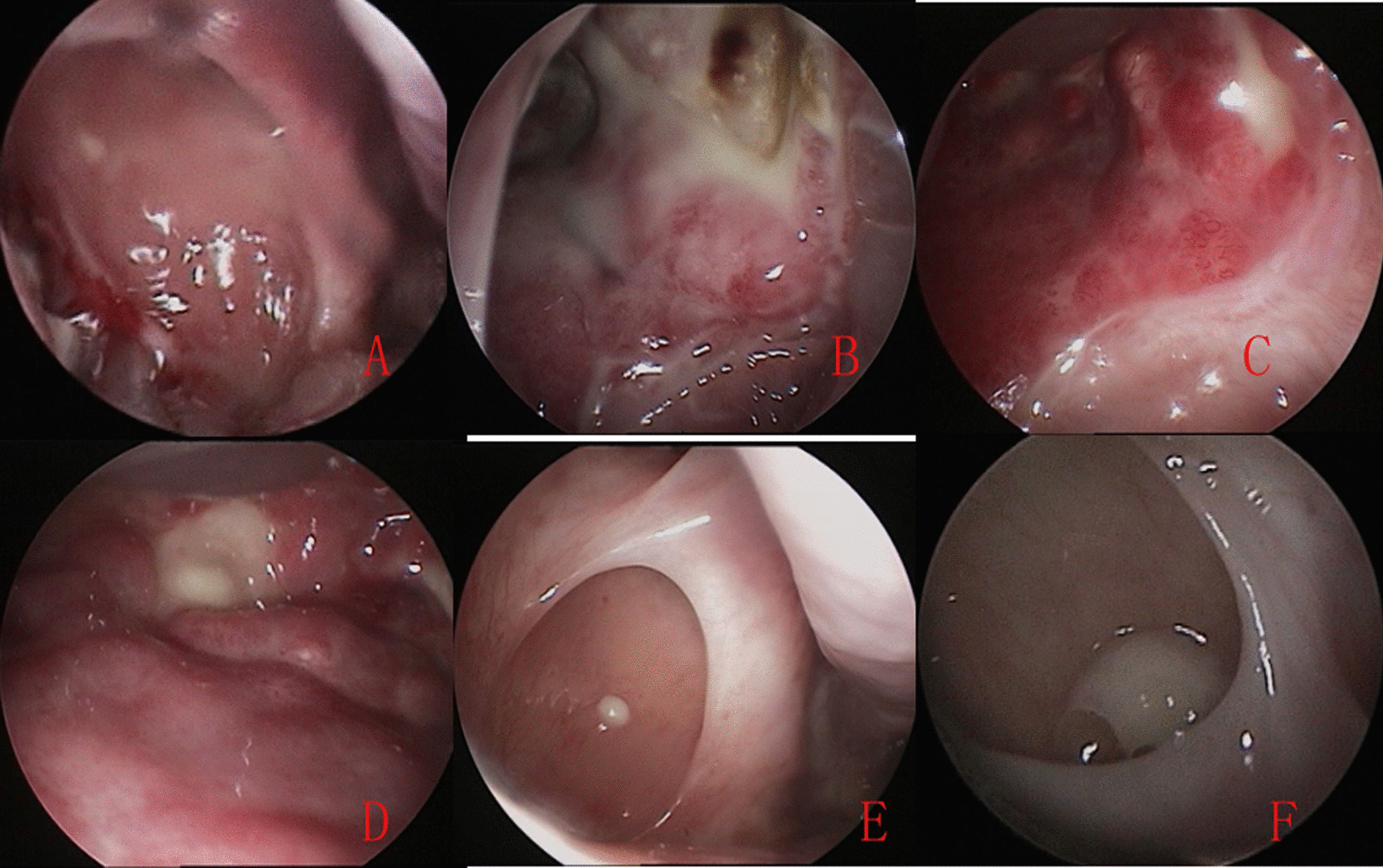
Persistent chronic inflammation in the inferlateral wall at second postoperative 4 months (**A**), mucosal inflammation gradually improved and mucosal epithelization formed, at third postoperative 2 months (**B**), 3 months (**C**), 4 months (**D**), 12 months (**E**), and 36 months (**F**). Figs. 1, 2 and 4 are the same patient

The average follow-up was 41 months (range, 37–61 months). CT and endoscope showed that tumor and symptom recurrence occurred in 2 patients (2.41%). All 2 patients had recurrence of benign IP, the time from surgery to recurrence was 7 and 13 months, respectively, that underwent one revision surgery. There wasn’t significant difference between revision surgery and primary surgery, also, significant difference wasn’t found between single and multifocal attachments. All 2 patients with recurrence originated from anterior medial wall and showed persistent chronic inflammation and failure epithelization in the MS following surgery (Fig. 4). In addition, although the opening of antrostomy was closed and CT revealed the uniform soft tissue shadow and hyperostosis of MS in 11(13.3%) patients, they didn’t report any symptoms and showed well epithelization of middle meatus mucosa.

## Discussion

The surgical management of choice for IPs involving the walls of the MS has historically consisted of a medial maxillectomy utilizing a lateral rhinotomy or a midface degloving approach [12]. With the development of endoscopical technique, endoscopic removal has been advocated as an effective, minimally invasive approach. However, endoscopic MMA alone didn’t usually expose the whole MS wall and completely eliminate all the tumor, thereby resulted in high recurrence rate of 16.6–52.2% [18–20]. Beswick et al. [21] demonstrated that, even with the combined use of shavers of different angles, only 81% of the surface area of the sinus could be reached via a large MMA. In current clinical practice these surgical approaches have been replaced in the last decade by two main techniques: endoscopic middle maxillectomy as the gold standard approach for IP arising from MS and open technique for tumors arising from the anterior wall of the MS, but the treatment of MS IPs remains a challenging issue and reached the recurrence rate of 4.5–10% [8, 9, 12].

Identification of the IP attachment site is vital for preventing the recurrence. Most of scholars reported that the attachment site may be accurately identified [11, 22]. In this study, although 28.9% had the history of previous surgery, MMA alone or Caldwell-Luc approach alone was performed in initial surgery, the attachment site hadn’t been eradicated, thus, the IP attachment sites were identified in all the patients. Wu et al. [22] also reported that the attachment site was identified in all 10 IPs with revision surgeries. The preferred treatment for IP is minimally invasive, attachment-oriented endoscopic surgery. This surgery includes the identification of the tumor attachment site, a subperiosteal dissection, and a resection or drilling of the underlying bone [1, 10, 11]. Despite the theoretical advantage of subperiosteal dissection and bone drill, these techniques have still shown high tumor recurrence rates. Healy et al. [20] reported a recurrence rate of 4.9% (3/61 patients) using tumor base bone drilling and 4.7% (1/21 patients) using cauterizing.

One possible reason is that IP could still exist in some bony crevices because of the irregularity of the bony surface although the underlying bone was drilled and led to recurrence. Chiu et al. [23] reported multiple bony crevices in all cases. In addition, some authors believed that overmuch drilling can puncture through the bone if the bony attachment is too thin and lead to possible cerebrospinal fluid leak, orbital injury, or other damage to underlying structures [1], thereby prevented further drilling of the underlying bone.

The correlation of the recurrence of IP and human papillomavirus (HPV) has been widely studied in recent but remained conflicting results [11, 24–27]. Some scholars believed that HPV infection is significantly associated with the recurrence of IP, especially for high-risk genotypes 16 and 18 [11, 24, 26, 27], while other found no correlation between risk of recurrence and HPV positivity [25]. Unfortunately, HPV testing was not performed in this study. In present study, MMA combined with different endoscopic approaches were applied. MMA alone was used to treat the IP with the single attachment site on the superior wall, whereas combined with EMM and/or Caldwell-Luc approach were respectively applied accordingly to the disease process for the IP with the attachment site on the rest of MS wall. MMA combined with endoscopic different approaches helped to clearly identify and reach the attachment sites. In addition, the ablation of the underlying bone was applied after mucosal stripping, periosteum ablation, bone drilling for all the patients, thereby effectively reduced the recurrence of IP. In this study, tumor recurrence occurred in 2 patients (2.41%) during follow-up of average 41 months. Theoretically, the four steps procedure completely eradicated the tumor, simultaneous well-organized bone drilling and ablation play the role of double insurance, thereby avoided the recurrence. Some scholars believed that similar to drilling, cauterization of the base is thought to destroy potential diseased epithelium embedded in the bone, which could reduce the recurrent tumor [20, 22].

In this study, the attachment site was in the anterior medial wall for all the 2 recurrent patients, IPs that originated from the anterior medial wall of the MS also could be the challenge. It could be due to that anterior medial portion can often just be a blind area when straight instruments are used. Similar to previous study [18], the multifocal attachments did not impact disease recurrence in this study. The recurrence rate wasn’t significantly different between revision surgery and primary surgery, also, significant difference wasn’t found between single and multifocal attachments. These findings suggested the absence of correlation between previous number of operations or attachment sites and IP recurrence, the key to prevent recurrence was whether to completely remove the tumor and underlying bone and margins.

Interestingly, the MS showed the persistent chronic inflammatory response and failure epithelization in 2 patients with recurrence in this study. It could be due to sustained release of inflammatory mediator by tumour cell. Some authors [28] suggested that high inflammatory cell population may help to predict IP recurrence or apparent malignant transformation. However, the correlation of IP recurrence and persistent inflammation need be further studied because of smaller sample in present study. Surprisingly, although the opening of antrostomy was closed and CT revealed the uniform soft tissue shadow and hyperostosis of MS in 11 (13.3%) patients, they didn’t report any symptoms and showed well epithelization of middle meatus mucosa.

## Conclusions

The four steps of operations of attachment sites of MS IP, including mucosal stripping, periosteum ablation, bone drilling and bone ablation, may effectively prevent the recurrence of MS IP.

## Data Availability

All data generated or analyzed during this study are included in the published article.
